# Learning curve of single-port laparoscopic appendectomy in a low-volume hospital

**DOI:** 10.3389/fsurg.2025.1630851

**Published:** 2025-11-20

**Authors:** Ji Yoon Jeong, Hyoung-Il Kim

**Affiliations:** 1 Department of Surgery, Armed Forces Yangju Hospital, Yangju, Republic of Korea; 2 Department of Surgery, Yonsei University College of Medicine, Seoul, Republic of Korea; 3 Gastric Cancer Center, Yonsei Cancer Center, Yonsei University Health System, Seoul, Republic of Korea

**Keywords:** acute appendicitis, laparoscopic appendectomy, single-port, learning curve, low-volume hospital

## Abstract

**Introduction:**

Single-port laparoscopic appendectomy (SPLA) has gained popularity, particularly among young patients, due to its cosmetic advantages. However, its adoption by surgeons in resource-constrained environments remains limited. This study assessed the learning curve of SPLA performed at a low-volume hospital (LVH).

**Methods:**

This retrospective study included patients who underwent appendectomy between May 2022 and December 2024 at a military hospital. A single surgeon newly initiated SPLA, while other surgeons performed conventional laparoscopic appendectomy (CLA). Clinical outcomes of SPLA were compared to CLA. The learning curve of SPLA was assessed using the moving average method and cumulative sum (CUSUM) analysis of the operation time.

**Results:**

Among 302 patients in the study, 125 underwent SPLA and 177 underwent CLA. The mean (range) age was 22.3 (18–49), similar in both groups. The mean (range) body mass index was 23.9 kg/m^2^ (16.9–34.9) in SPLA, 24.2 kg/m^2^ (17.2–36.8) in CLA. There were no significant differences in the mean operation time or postoperative surgical complication rates between SPLA and CLA (Operation time: SPLA 48.6 min vs. CLA 47.1 min, *P* = 0.582; Postoperative surgical complication rate: SPLA 4.8% vs. CLA 5.1%, *P* = 0.911). Based on a two-breakpoint regression of the CUSUM of SPLA operation time, three learning phases were divided: the initial phase (1st–13th cases), the competent phase (14th–36th cases), and the mastery phase (37th–125th cases). The mean operation time significantly decreased throughout the phases (70.6 min vs. 52.0 min vs. 44.5 min, *P* < 0.001). A trend toward reduced use of additional rescue analgesics and postoperative complication rates in the mastery phase was observed but did not reach statistical differences (Additional rescue analgesics: 38.5% vs. 39.1% vs. 20.2%, *P* = 0.068; Postoperative complication rate: 15.4% vs. 4.3% vs. 3.4%, *P* = 0.101).

**Conclusion:**

The learning curve for the SPLA in a low-volume hospital consisted of three phases. The competent phase was achieved after the 13th case, and the mastery after the 36th. With sufficient prior laparoscopic experience, surgeons may safely adopt SPLA in low-volume settings, particularly when initiated in young, healthy, and non-obese patients.

## 1 Introduction

Single-port laparoscopic appendectomy (SPLA) is gaining popularity for its advantages, including reduced postoperative pain, faster recovery, and improved cosmetic outcomes, which are particularly appealing to young patients (1, 2). However, despite these benefits, the adoption of SPLA in resource-constrained environments remains limited. Surgeons in these settings often face challenges such as limited experience with single-port techniques, lack of supervision, or restricted access to specialized instruments.

Previous studies have reported that hospital volume is closely associated with appendectomy outcomes, but whether low-volume hospitals (LVH) can safely adopt SPLA remains uncertain (3–5). Although previous studies have demonstrated the feasibility of SPLA in LVHs, the small sample sizes of those studies, which were fewer than 40 patients, and the lack of learning curve analysis leave critical gaps in understanding the practical application of SPLA (6, 7). The surgeon's learning curve, the time and number of cases required to achieve proficiency, is a key factor influencing the successful implementation of SPLA (8). Understanding this learning process is essential for facilitating the transition from multiport to single-port techniques, particularly in resource-limited settings.

Therefore, this study aimed to assess the learning curve for SPLA in an LVH, providing insights into their feasibility, safety, and effectiveness in resource-constrained environments.

## 2 Materials and methods

### 2.1 Patients and study design

This retrospective cohort study included patients who were diagnosed with acute appendicitis and underwent laparoscopic appendectomy at Armed Forces Yangju Hospital between May 2022 and December 2024.

**Figure 1:**
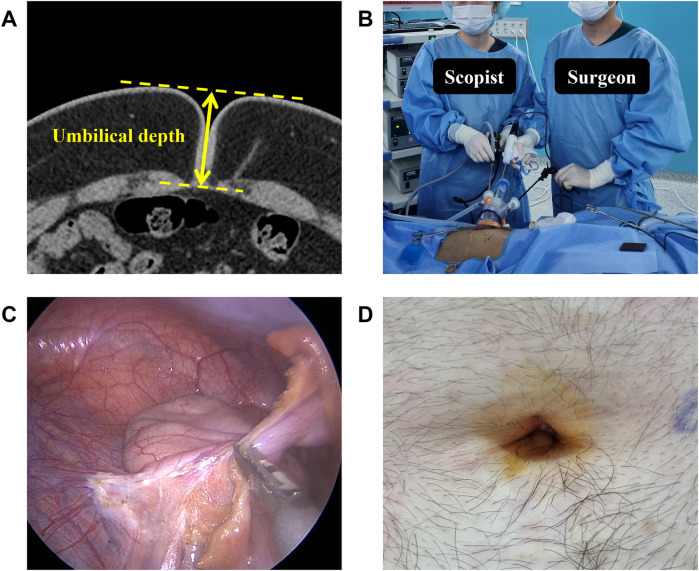
Single-port laparoscopic appendectomy. **(A)** Umbilical depth was measured in preoperative CT. **(B)** Operator stood to the left of the scopist and used only straight, rigid laparoscopic instruments. **(C)** Ultrasonic surgical devices were used to resect the mesoappendix and ligate the appendiceal artery. **(D)** Umbilical wound on postoperative day 3.

Acute appendicitis was diagnosed using abdominal computed tomography (CT) scans. The severity of appendicitis was graded as simple, suppurative, periappendiceal abscess, or perforated based on the preoperative CT findings. Umbilical depth on CT was measured from the peritoneal level to the highest level of the skin surface adjacent to the umbilicus (Figure 1A).

This institution performs fewer than 120 laparoscopic surgeries annually by four surgeons, primarily appendectomies. There are no specialized surgical assistants, and one of the circulating nurses rotates randomly to act as the scopist. Conventional laparoscopic appendectomy (CLA) has been the standard treatment in Korean Armed Forces Hospitals. However, a single surgeon introduced SPLA to the Armed Forces Yangju Hospital, while other surgeons continued performing CLA. This surgeon completed four years of residency and one year of fellowship training at a tertiary hospital but had no prior experience in single-port laparoscopic surgery.

Patients were allocated to SPLA or CLA based on both patient preference and the surgeon's clinical judgment. In the early period, patients with severe inflammation (periappendiceal abscess or perforated appendicitis) were treated with CLA, while SPLA was offered to patients with simple or suppurative appendicitis. Patients who declined SPLA also underwent CLA. As the surgeon's experience increased, the indication for SPLA gradually expanded to more advanced inflammation.

The clinical outcomes of patients undergoing SPLA were compared with those undergoing CLA. The learning curve was assessed based on the outcomes of SPLA. Clinical data from electronic medical records were retrospectively reviewed. The study was approved by the Institutional Review Board of Armed Forces Medical Command (2024-01-009), which waived the requirement for informed consent because of its retrospective nature.

### 2.2 Surgical procedures

All patients received a dose of preoperative empiric antibiotics (intravenous ceftriaxone 2 g) 30 min before the incision was made. SPLA was performed in the following three procedures.

In the opening procedure, the patient was placed in a supine position. A 15–20 mm transumbilical incision was made using the open Hasson technique. A single-port device (Lemon Single, Islemon, South Korea) was inserted, and a pneumoperitoneum was created.

In the appendectomy procedure, the operator stood to the left of the scopist, and used only straight rigid laparoscopic instruments, including a 5 mm scope with a 30-degree angle (Figure 1B). The mesoappendix and the appendiceal artery were resected using an ultrasonic surgical device (Figure 1C). The appendix base was ligated with two Endo-loops (GEMSLOOP-PDO, GEMS, South Korea). The specimens were extracted directly through the port with a grasper during the first 41 cases. After the 42nd case, specimen retrieval bags were used to reduce contamination risk. No surgical drains were placed. If necessary, an additional 5 mm trocar was inserted during surgery in the left lower quadrant area.

In the closure procedure, the fascia was closed with three interrupted sutures, including a figure-of-eight suture at the center. Care was taken to ensure the proper fascia closure to prevent incisional hernia. Both ends of the subcutaneous layer were sutured to minimize dead space. Dermis sutures were completed (Figure 1D).

CLA was performed using the 3-port method. A 12 mm trocar was inserted at the subumbilical area using the open Hasson technique. Additional two 5 mm trocars were placed in the suprapubic and left lower quadrants. The appendectomy followed the same method as in SPLA. The subumbilical fascia was closed, whereas the fascia of the 5 mm port sites was left unclosed. The skin was closed using either a skin stapler or a nylon suture.

### 2.3 Postoperative management

Patients were allowed to sip water 8 h after surgery, followed by a solid diet on the next day, regardless of flatus. The progression of the diet was adjusted based on the patient's condition and the surgeon's judgment. Postoperative antibiotics (intravenous ceftriaxone 2 g) were administered the day after surgery if the operative findings indicated a contaminated field. Routine preemptive analgesics (intravenous acetaminophen 1 g) were given immediately after surgery and again before sleep, with oral analgesics starting the following day.

Given the patient's status as a soldier, discharge was generally scheduled after more than seven postoperative days (POD). Patients underwent open follow-up after discharge. For those who requested earlier discharge, it was permitted only after confirming adequate pain control and diet tolerance through physical examination. Those discharged before seven POD underwent follow-up within 1–2 weeks after discharge.

### 2.4 Postoperative outcome

Postoperative pain was assessed based on the number of rescue analgesics administered (9, 10). Additional rescue analgesics (intravenous ketorolac 30 mg) were given when patients reported pain with a numerical rating scale score of 4 or higher, with a minimum interval of 4 h between doses (11).

Postoperative surgical complications were graded according to Clavien-Dindo classification (12). The overall postoperative surgical complication rate was defined as Clavien-Dindo grade I or higher. Wound dehiscence referred to superficial skin separation or serosal discharge from the subcutaneous layer, since fascia dehiscence was not reported in overall patients.

Readmissions related to appendectomy were recorded without a limit on duration.

### 2.5 Learning curve and statistical analysis

The operation time was analyzed using three methods to assess the learning curve of SPLA.

First, the moving average method smooths short-term fluctuations in operation time, highlighting long-term trends. A moving average order of 10 was used.

Second, cumulative sum (CUSUM) analysis presents the sequential difference between individual operation time and the mean operation time. It is calculated by the following equation, where $x_{i}$ is the individual operation time, and μ is the mean of the overall operation time.

$$\mathrm{CUSUM}=\overset{n}{\underset{i=1}{\sum }}\;(x_{i}-\mu )$$

Third, piecewise linear analysis detects changes in trends by dividing the data into distinct segments. A two-breakpoint regression model was applied to the CUSUM results.

IBM SPSS Statistics for Windows, version 27.0 (IBM Corp., Armonk, NY, USA) was used for statistical analyses. The piecewise linear regression analysis was conducted using the R statistical package (version 4.4.3). Categorical variables are presented as numbers and percentages, and were analyzed by the chi-square test, Fisher's exact test, or linear-by-linear test. Continuous variables are presented as the means and ranges, and were analyzed by one-way analysis of variance (ANOVA) among groups. Univariate analysis was performed to identify potential preoperative clinical factors associated with the operation time of SPLA. A Student's *t*-test was used for binary variables, ANOVA for variables with three or more categories, and a correlation analysis with Spearman's test for continuous variables. Factors with a *P*-value of less than 0.10 in univariate analysis were subsequently included in multiple linear regression analyses. A *P*-value of less than 0.05 was considered statistically significant.

## 3 Results

### 3.1 Patient demographics and outcomes

**Figure 2:**
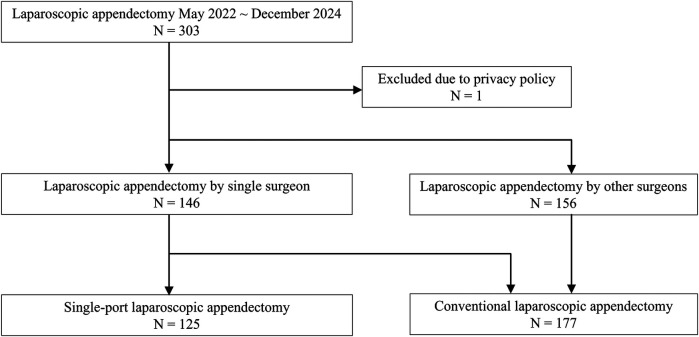
Study profile.

Among 303 patients who underwent laparoscopic appendectomy between May 2022 and December 2024, one patient who requested a privacy policy for clinical records was excluded. A total of 125 patients who underwent SPLA and 177 patients who underwent CLA were included in the analysis (Figure 2).

Patient demographics and clinical characteristics are summarized in Table 1. The mean (range) age was 22.3 (18–49), similar in both groups. The mean (range) body mass index was 23.9 kg/m^2^ (16.9–34.9) in SPLA, 24.2 kg/m^2^ (17.2–36.8) in CLA. There were no statistically significant differences in the severity of appendicitis between SPLA and CLA groups (*P* = 0.862). The mean operation time showed no significant differences between the two groups (SPLA 48.6 min vs. CLA 47.1 min, *P* = 0.582). However, SPLA was associated with significantly less blood loss and an earlier solid diet start (Blood loss: SPLA 4.4 mL vs. CLA 6.3 mL, *P* = 0.027; POD of solid diet start: SPLA 1.1 vs. CLA 1.3, *P* < 0.001). The overall postoperative surgical complication rates did not differ significantly (SPLA 4.8% vs. CLA 5.1%, *P* = 0.911).

**Table 1:** Patient demographics and clinical characteristics .

Characteristics	SPLA (*n* = 125)	CLA (*n* = 177)	*P*-value
Sex (%)			0.169
Male	122 (97.6%)	176 (99.4%)	
Female	3 (2.4%)	1 (0.6%)	
Age (range)	22.3 (18–49)	22.3 (18–49)	0.974
Body mass index (kg/m^2^, range)	23.9 (16.9–34.9)	24.2 (17.2–36.8)	0.594
Umbilical depth in CT (mm, range)	22.6 (7.2–53.3)	21.4 (8.1–43.5)	0.187
Preoperative fever (%)			0.206
No	111 (90.2%)	151 (85.3%)	
Yes	12 (9.8%)	26 (14.7%)	
Inflammation marker			
WBC count (10^3^ μL, range)	12.37 (44.70–24.80)	12.92 (40.60–23.65)	0.283
C-reactive protein (mg/dL, range)	1.23 (0.03–11.36)	2.45 (0.01–28.86)	0.003
Severity in preoperative CT (%)			0.862
Simple	68 (54.4%)	89 (50.3%)	
Suppurative	49 (39.2%)	74 (41.8%)	
Periappendiceal abscess	6 (4.8%)	9 (5.1%)	
Perforated	2 (1.6%)	5 (2.8%)	
Type of appendix location (%)			0.933
Retrocecal	20 (16.0%)	29 (16.5%)	
Pelvic	74 (59.2%)	96 (54.5%)	
Sub-cecal	13 (10.4%)	22 (12.5%	
Post-ileal	16 (12.8%)	25 (14.2%)	
Pre-ileal	2 (1.6%)	4 (2.3%)	
Operation time (min, range)	48.6 (25–133)	47.1 (12–180)	0.582
Blood loss (mL, range)	4.4 (1–50)	6.3 (1–100)	0.027
First flatus (POD, range)	0.6 (0–1)	0.7 (0–1)	0.058
Solid diet start (POD, range)	1.1 (1–4)	1.3 (1–10)	<0.001
Additional rescue analgesic (%)			0.115
None	93 (74.4%)	127 (71.8%)	
One	24 (19.2%)	38 (21.5%)	
Twice	8 (6.4%)	6 (3.4%)	
More than three	0	6 (3.4%)	
Discharge (POD, range)	6.6 (1–22)	6.9 (1–28)	0.450
Postoperative surgical complication (%)	6 (4.8%)	9 (5.1%)	0.911
CDC Grade I	5 (4.0%)	5 (2.8%)	
CDC Grade II	0	1 (0.6%)	
CDC Grade IIIb	1 (0.8%)	3 (1.7%)	
Readmission (%)	3 (2.4%)	1 (0.6%)	0.310

SPLA, single-port laparoscopic appendectomy; CLA, conventional laparoscopic appendectomy; CT, computed tomography; POD, postoperative day; CDC, Clavien-Dindo classification.

**Figure 3:**
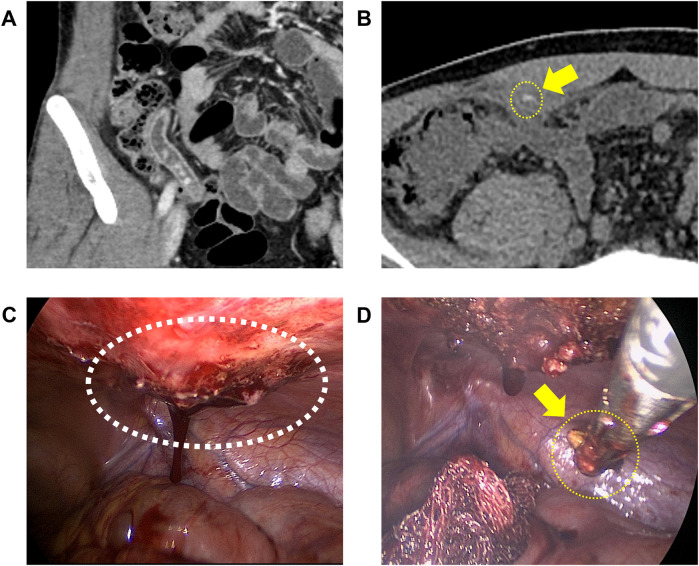
The patient who underwent reoperation. The yellow arrow indicates the dropped appendicolith. **(A)** Multiple appendicoliths were identified before the initial appendectomy. **(B)** CT on postoperative day 20 revealed a 20 mm intra-abdominal abscess with a 2 mm appendicolith inside. **(C)** Findings of laparoscopic view during reoperation. The white circle indicates an intra-abdominal abscess surrounded by granulation tissue at the abdominal wall. **(D)** Removing appendicolith after the excision of granulation tissue.

In the SPLA group, wound dehiscence occurred in five patients (4.0%). Two patients (1.6%) required readmission and bedside wound repair, while the other three (2.4%) were treated with simple dressing. One patient (0.8%) underwent reoperation due to an intra-abdominal abscess and a dropped appendicolith. This occurred in the 39th case, where the patient was initially discharged on POD 4 and readmitted on POD 20 due to aggravation of abdominal pain. The CT revealed a 20 mm intra-abdominal abscess with a dropped 2 mm appendicolith inside. As the abdominal pain was not relieved with antibiotic treatment, laparoscopic exploration and removal of the appendicolith were performed on POD 34 (Figure 3).

In the CLA group, wound dehiscence occurred in five patients (2.8%), all managed with simple dressing. One patient (0.6%) developed postoperative ileus on POD 3, which improved with nasogastric tube insertion and fluid therapy. Three patients (1.7%) underwent reoperation after CLA, two patients (1.1%) for intra-abdominal abscess, and one patient (0.6%) for postoperative bleeding.

### 3.2 Learning curve of SPLA

**Figure 4:**
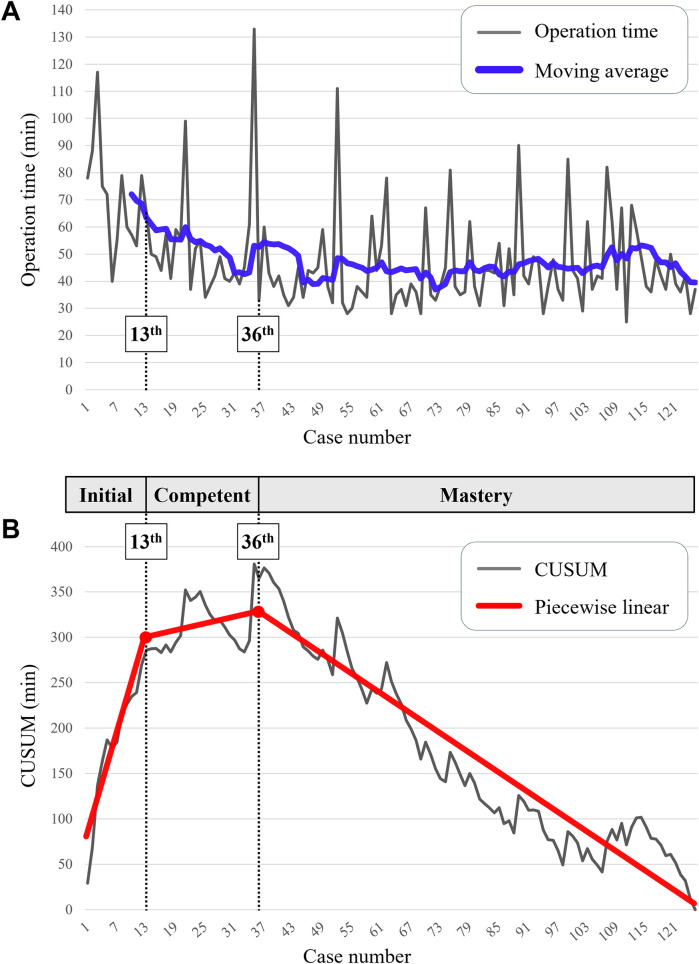
Learning curve. **(A)** Operation time and trendlines. The blue trendline indicates a moving average in order of 10. **(B)** Cumulative sum (CUSUM) of operation time. Piecewise linear analysis of CUSUM identified two breakpoints at the 13th and 36th cases (statistically estimated at 13.3 and 36.0, respectively). Based on these points, the learning curve was divided into three phases (initial, competent, mastery).

The moving average method was applied to the operation time of SPLA to visualize trends (Figure 4A). In the piecewise linear analysis of CUSUM, two breakpoints were identified (adjusted *R*^2^ = 0.918). The first breakpoint was at 13.3 (95% confidence interval, 10.9–15.7) and the second was at 36.0 (95% confidence interval, 29.9–42.1) (Figure 4B). Based on these breakpoints, three learning phases were divided: the initial phase (1st–13th), the competent phase (14th–36th), and the mastery phase (37th–125th).

In the mastery phase, the proportions of the average time consumed for each step of the SPLA were as follows: opening of the abdominal wall, 17.3% (mean 7.7 min, range 3–29); appendectomy, 50.3% (mean 22.4 min, range 9–90); and closure of the abdominal wall, 32.4% (mean 14.4 min, range 5–42).

Patient characteristics and postoperative outcomes of SPLA were compared across the three phases (Table 2). Although the inclusion of SPLA expanded to appendicitis with periappendiceal abscess or perforated appendicitis in the later phases, there was no significant difference in the severity of appendicitis across the phases (*P* = 0.119). Four patients (3.2%) required additional trocars, and no open conversions were necessary for the overall phases. The mean operation time significantly decreased throughout the phases (70.6 min vs. 52.0 min vs. 44.5 min, *P* < 0.001). There was a trend toward reduced use of additional rescue analgesics and postoperative surgical complication rates in the mastery phase, but there were no statistical differences (Additional rescue analgesics: 38.5% vs. 39.1% vs. 20.2%, *P* = 0.068; Postoperative surgical complication rate: 15.4% vs. 4.3% vs. 3.4%, *P* = 0.101).

**Table 2:** Outcomes of single-port laparoscopic appendectomy according to the learning phases.

Outcomes	Initial phase (1st–13th) *n* = 13	Competent phase (14th–36th) *n* = 23	Mastery phase (37th–125th) *n* = 89	*P*-value
Body mass index (kg/m^2^, range)	22.8 (19.2–27.1)	23.2 (18.8–28.1)	24.3 (16.9–34.9)	0.131
Umbilical depth in CT (mm, range)	22.9 (12.6–36.5)	21.8 (13.5–37.2)	22.8 (7.2–53.3)	0.852
Severity in preoperative CT (%)				0.119
Simple	3 (23.1%)	12 (52.2%)	53 (59.6%)	
Suppurative	10 (76.9%)	9 (43.5%)	30 (33.7%)	
Peri-appendiceal abscess	0	1 (4.3%)	5 (5.6%)	
Perforated	0	1 (4.3%)	1 (1.1%)	
Additional trocar (%)	0	1 (4.3%)	3 (3.4%)	0.742
Operation time (min, range)	70.6 (40–117)	52.0 (33–133)	44.5 (25–111)	<0.001
Blood loss (mL, range)	4.5 (2–15)	4.7 (2–30)	4.3 (1–50)	0.930
Additional rescue analgesic (%)				0.068
None	8 (61.5%)	14 (60.9%)	71 (79.8%)	
Once	4 (30.8%)	6 (26.1%)	14 (15.7%)	
Twice	1 (7.7%)	3 (13.0%)	4 (4.5%)	
Postoperative surgical complication (%)	2 (15.4%)	1 (4.3%)	3 (3.4%)	0.101
CDC Grade I	2 (15.4%)	1 (4.3%)	2 (2.2%)	
CDC Grade IIIb	0	0	1 (1.1%)	

CT, computed tomography; CDC, Clavien-Dindo classification.

### 3.3 Factors associated with SPLA operation time

Univariate analysis identified that the learning phase, body mass index, umbilical depth, and severity of appendicitis on preoperative CT were potentially associated with the operation time of SPLA (Table 3). Multiple linear regression analysis confirmed that the learning phase, umbilical depth, and severity of appendicitis on preoperative CT were significantly associated factors, whereas body mass index was not (Overall regression *P* < 0.001, adjusted *R*^2^ = 0.468, Durbin-Watson = 1.981) (Table 4).

**Table 3:** Univariate analysis of preoperative clinical factors and operation time in single-port laparoscopic appendectomy.

Preoperative clinical factor	Test statistic			*P*-value
	Value		95% CI	
Age^a^	Spearman's *ρ*	−0.024	−0.198 to 0.145	0.789
Body mass index^a^	Spearman's ρ	0.167	−0.018 to 0.333	**0**.**063**
Umbilical depth in CT^a^	Spearman's ρ	0.332	0.154 to 0.491	**<0**.**001**
White blood cell count^a^	Spearman's ρ	−0.004	−0.176 to 0.174	0.962
Sex^b^	T(p)	−1.615	N/A	0.109
Preoperative fever^b^	T(p)	−1.321	N/A	0.212
Learning phase^c^	F	13.396	N/A	**<0.001**
Severity in preoperative CT^c^	F	22.733	N/A	**<0.001**
Type of appendix location^c^	F	0.141	N/A	0.967

CI, confidence interval; CT, computed tomography; N/A, not applicable.

Bold indicates statistically significant *P*-values.

a Correlation analysis.

b Student's *t*-test.

c One-way analysis of variance.

**Table 4:** Multiple linear regression of preoperative clinical factors and operation time in single-port laparoscopic appendectomy.

Preoperative clinical factor	Unstandardized coefficient		95% CI for B	Standardized coefficient	T(*p*)	*P*-value	VIF
	B	SE					
Learning phase	−10.613	1.915	−14.404 to −6.823	−0.375	−5.544	**<0** **.** **001**	1.064
Body mass index	−0.438	0.547	−1.522 to 0.645	−0.078	−0.801	0.425	2.203
Umbilical depth in CT	0.907	0.227	0.457 to 1.357	0.381	3.990	**<0** **.** **001**	2.127
Severity in preoperative CT	9.313	1.310	6.719 to 11.907	0.468	3.990	**<0** **.** **001**	1.010

Overall regression *P* < 0.001, F = 28.245, Adjusted *R*^2^ = 0.468, Durbin-Watson = 1.981.

SE, standard error; CI, confidence interval; VIF, variance inflation factor; CT, computed tomography.

Bold indicates statistically significant *P*-values.

## 4 Discussion

In this retrospective study, the learning curve for SPLA in an LVH presented three phases. The competent phase was achieved after the 13th case, and the mastery phase was achieved after the 36th case. The overall surgical outcomes of SPLA were comparable to CLA.

Although previous studies have supported the feasibility and safety of SPLA (1, 13–15), its adoption in resource-constrained environments, such as LVHs, remains uncertain. Korean Armed Forces Hospitals, which are national healthcare institutions with low surgical volumes, face barriers to adopting advanced surgical techniques. These include a lack of experienced assistants or supervisors and limited access to high-cost instruments like articulated devices. Despite these constraints, the findings in this study demonstrate the feasibility of introducing SPLA in such an environment. The mean operation time of 48 min and surgical complication rate of 4.8% for SPLA were comparable to those of CLA, which aligns with previous studies that supported the feasibility and safety of SPLA (2, 16, 17).

During the initial phase of SPLA, CUSUM analysis showed a positive slope up to the 13th case, indicating the surgeon's adaptation to the technical demands of SPLA, which contributed to longer operation times. After that point, CUSUM showed repeated fluctuations with a less pronounced positive slope, indicating growing familiarity during the competent phase. The slope of CUSUM turned consistently negative after the 36th case, reflecting improved proficiency during the mastery phase. Previous meta-analyses raised concerns that the learning curve for SPLA may be complex, based on studies suggesting that approximately 30 cases are needed to achieve adequate surgical skills and 90 cases to gain advanced proficiency (18, 19). While earlier studies involved the initial application of SPLA to complicated appendicitis, attempting new techniques in challenging cases without experience could lead to a prolonged learning process or high morbidity in unsupervised settings. In contrast, SPLA was initially applied to uncomplicated appendicitis in this study and gradually expanded to more complex cases as the surgeon's experience accumulated. This resulted in a clearer and less complex learning curve that may reflect a more practical progression for resource-constrained settings. Nevertheless, the number of breakpoints identified in this study should be interpreted as surgeon-specific reference points rather than general thresholds, since it may vary across surgeons depending on prior experience and institutional resources.

Despite reaching a plateau in operation time during the mastery phase, some fluctuations persisted. Multiple linear regression identified preoperative factors, such as appendicitis severity and umbilical depth, as significant contributors to operation time. The severity of appendicitis is a well-known factor that prolongs surgery (20). Interestingly, umbilical depth emerged as a more relevant predictor than body mass index. Since SPLA relies on a transumbilical approach, a deeper umbilicus may increase technical difficulty during abdominal wall opening and closure, accounting for more than half of the total operation time. Although previous studies have debated the impact of body mass index on SPLA operation time, this study suggests that a simple measurement of umbilical depth on CT provides a better predictor (7, 19, 21–23). Accordingly, SPLA may be technically easier and more suitable for initial adoption in non-obese patients with a shallow umbilicus, in whom the transumbilical approach is more straightforward.

Improvements in surgical outcomes were observed in later phases, including trends toward reduced use of rescue analgesics and fewer complications. Shorter incisions in later phases likely contributed to faster abdominal wall procedures, reduced postoperative pain, and wound complications. In addition, a specimen retrieval bag was introduced after an incident involving a dropped appendicolith in the 41st case, significantly reducing the risk of postoperative infection (24). It is unclear whether the use of specimen bags reduced wound dehiscence because of the retrospective nature of this study. However, these refinements demonstrate evolving surgical competence and the integration of conventional laparoscopic principles to enhance surgical outcomes.

The patient cohort in this study consisted predominantly of young, healthy male soldiers, reflecting the demographics of military hospitals. While this homogeneity may limit generalizability to broader populations, it also offers methodological advantages for assessing the pure technical learning process. The relatively uniform characteristics of this population, such as young and healthy, minimized variability related to patient comorbidities, allowing a clearer interpretation of surgeon-related learning rather than patient-related factors. Moreover, younger patients tend to have higher cosmetic expectations, and SPLA provides distinct aesthetic advantages through a single hidden umbilical incision (25). Therefore, while this homogeneous population limits external applicability, it simultaneously enhances internal validity and offers clinically relevant insights for younger patients who particularly value cosmetic outcomes.

Cost analysis was not performed in this study because the Korean Armed Forces Hospitals provide emergency care to soldiers free of charge. However, previous studies showed that SPLA did not increase overall costs compared with CLA (26–28). As SPLA replaces three conventional trocars with a single multi-channel port without additional specialized instruments, a cost analysis in this study would likely have shown similar results. In cases requiring an additional trocar, the procedural cost might have been slightly higher. However, such cases were uncommon (overall 3%) and likely had a minimal impact on overall cost. Accordingly, SPLA appears both technically and economically feasible even in low-resource environments.

Identifying approximate checkpoints for achieving competency and mastery helps establish standards and facilitate the transition from CLA to SPLA in diverse healthcare settings. Although this study was conducted in a homogenous military population, the findings provide useful reference points for hospitals with similar demographic or resource profiles. In settings with different demographics, these findings may help identify which patient groups are most suitable for the initial implementation of SPLA. Therefore, SPLA can be safely initiated in low-volume or resource-limited settings with appropriate patient selection—beginning with young, healthy, and non-obese patients—and gradually expanded as surgical experience accumulates.

This study has several limitations. First, the SPLA data are derived from a single surgeon, which limits generalizability. The surgeon's prior experience in a tertiary hospital during training may have influenced the postoperative outcomes and learning curve. Additionally, because most patients in military hospitals are young males, potential selection bias may exist, further limiting the applicability of this study to the general population. However, despite its limitations, such a homogenous population allowed a clearer assessment of the surgeon's technical learning process. Future studies involving multiple surgeons from various centers could provide a more representative learning curve for SPLA. Second, this was a retrospective study without randomization. The surgeon involved in the learning curve often performed CLA in severe inflammation, which could introduce selection bias. Thus, the learning curve may not fully represent the challenges of SPLA for severe appendicitis or technically demanding scenarios. Third, this study did not assess long-term outcomes of SPLA. A previous study reported that single-port laparoscopic surgeries carry a risk of incisional hernia, with an incidence of 2.9% at 36 months of follow-up (29). Although no incisional hernia was observed during the short follow-up in this study, the true incidence remains uncertain because most patients were lost to follow-up after relocation or discharge from military service. Future prospective studies with long-term surveillance are required to clarify the durability and safety of SPLA in LVHs.

## 5 Conclusion

The learning curve for SPLA in a low-volume hospital showed three phases. The competent phase was achieved after the 13th case, and mastery after the 36th. Postoperative complication rates remained acceptable throughout the learning phases, and overall outcomes were comparable to CLA. With sufficient prior laparoscopic experience, surgeons in low-volume settings can safely adopt SPLA with acceptable efficiency. Considering that young, healthy, and non-obese patients often have higher cosmetic demands and fewer comorbidities, SPLA may be best introduced initially in such populations. Its indications can be gradually expanded to more diverse patient groups as surgical experience accumulates.

## Data Availability

The datasets presented in this article are not readily available due to privacy and ethical restrictions. Requests to access the datasets should be directed to Ji Yoon Jeong, jjy91007@naver.com

## References

[1] Aly OE Black DH Rehman H Ahmed I. Single incision laparoscopic appendicectomy versus conventional three-port laparoscopic appendicectomy: a systematic review and meta-analysis. Int J Surg. (2016) 35:120–8. 10.1016/j.ijsu.2016.09.087 27686264

[2] Lee WS Choi ST Lee JN Kim KK Park YH Lee WK Single-port laparoscopic appendectomy versus conventional laparoscopic appendectomy: a prospective randomized controlled study. Ann Surg. (2013) 257(2):214–8. 10.1097/SLA.0b013e318273bde4 23241869

[3] Murata A Mayumi T Muramatsu K Ohtani M Matsuda S. Effect of hospital volume on outcomes of laparoscopic appendectomy for acute appendicitis: an observational study. J Gastrointest Surg. (2015) 19(5):897–904. 10.1007/s11605-015-2746-y 25595310

[4] O'Connell RM Abd Elwahab S Mealy K. The impact of hospital grade, hospital-volume, and surgeon-volume on outcomes for adults undergoing appendicectomy. Surgeon. (2020) 18(5):280–6. 10.1016/j.surge.2019.10.006 31806483

[5] Cawich SO Mohanty SK Simpson LK Ramdass MJ Naraynsingh V. Is laparoscopic appendectomy safe when performed in a low volume setting? Int J Biomed Sci. (2014) 10(1):31–5. 10.59566/IJBS.2014.10031 24711747 PMC3976445

[6] Kang BH Yoon KC Jung SW Lee GR Lee HS. Feasibility of single-incision laparoscopic appendectomy in a small hospital. Ann Surg Treat Res. (2016) 91(2):74–9. 10.4174/astr.2016.91.2.74 27478812 PMC4961889

[7] Lee J Baek J Kim W. Laparoscopic transumbilical single-port appendectomy: initial experience and comparison with three-port appendectomy. Surg Laparosc Endosc Percutan Tech. (2010) 20(2):100–3. 10.1097/SLE.0b013e3181d84922 20393336

[8] Hopper AN Jamison MH Lewis WG. Learning curves in surgical practice. Postgrad Med J. (2007) 83(986):777–9. 10.1136/pgmj.2007.057190 18057179 PMC2750931

[9] Kinoshita J Fushida S Kaji M Oyama K Fujimoto D Hirono Y A randomized controlled trial of postoperative intravenous Acetaminophen plus thoracic epidural analgesia vs. thoracic epidural analgesia alone after gastrectomy for gastric cancer. Gastric Cancer. (2019) 22(2):392–402. 10.1007/s10120-018-0863-5 30088162 PMC6394709

[10] Wick EC Grant MC Wu CL. Postoperative multimodal analgesia pain management with nonopioid analgesics and techniques: a review. JAMA Surg. (2017) 152(7):691–7. 10.1001/jamasurg.2017.0898 28564673

[11] Breivik H Borchgrevink PC Allen SM Rosseland LA Romundstad L Hals EK Assessment of pain. Br J Anaesth. (2008) 101(1):17–24. 10.1093/bja/aen103 18487245

[12] Dindo D Demartines N Clavien PA. Classification of surgical complications: a new proposal with evaluation in a cohort of 6336 patients and results of a survey. Ann Surg. (2004) 240(2):205–13. 10.1097/01.sla.0000133083.54934.ae 15273542 PMC1360123

[13] Han Y Yuan H Li S Wang WF. Single-incision versus conventional three-port laparoscopic appendectomy for acute appendicitis: a meta-analysis of randomized controlled trials. Asian J Surg. (2024) 47(2):864–73. 10.1016/j.asjsur.2023.12.179 38185558

[14] Moriguchi T Machigashira S Sugita K Kawano M Yano K Onishi S A randomized trial to compare the conventional three-port laparoscopic appendectomy procedure to single-incision and one-puncture procedure that was safe and feasible, even for surgeons in training. J Laparoendosc Adv Surg Tech A. (2019) 29(3):392–5. 10.1089/lap.2018.0195 30418099

[15] Suh SG Sohn HJ Kim BG Park JM Choi YS Park YK Single-Incision laparoscopic appendectomy by surgical trainees. Surg Laparosc Endosc Percutan Tech. (2016) 26(6):470–2. 10.1097/SLE.0000000000000340 27846185 PMC5142366

[16] Carter JT Kaplan JA Nguyen JN Lin MY Rogers SJ Harris HW. A prospective, randomized controlled trial of single-incision laparoscopic vs conventional 3-port laparoscopic appendectomy for treatment of acute appendicitis. J Am Coll Surg. (2014) 218(5):950–9. 10.1016/j.jamcollsurg.2013.12.052 24684867

[17] Frutos MD Abrisqueta J Lujan J Abellan I Parrilla P. Randomized prospective study to compare laparoscopic appendectomy versus umbilical single-incision appendectomy. Ann Surg. (2013) 257(3):413–8. 10.1097/SLA.0b013e318278d225 23386239

[18] Cirocchi R Cianci MC Amato L Properzi L Buononato M Di Rienzo VM Laparoscopic appendectomy with single port vs conventional access: systematic review and meta-analysis of randomized clinical trials. Surg Endosc. (2024) 38(4):1667–84. 10.1007/s00464-023-10659-w 38332174 PMC10978699

[19] Kim Y Lee W. The learning curve of single-port laparoscopic appendectomy performed by emergent operation. World J Emerg Surg. (2016) 11:39. 10.1186/s13017-016-0096-z 27499804 PMC4975885

[20] Alotaibi AM Alfawaz M Felemban L Moshref L Moshref R. Complicated appendicitis increases the hospital length of stay. Surg Open Sci. (2022) 9:64–8. 10.1016/j.sopen.2022.05.006 35692621 PMC9178463

[21] Ma L Pan H Chen K. Impact of visceral obesity on the short-term outcomes after laparoscopic appendectomy. Scand J Gastroenterol. (2023) 58(7):757–63. 10.1080/00365521.2023.2173022 36728716

[22] Litz CN Farach SM Danielson PD Chandler NM. Obesity and single-incision laparoscopic appendectomy in children. J Surg Res. (2016) 203(2):283–6. 10.1016/j.jss.2016.03.039 27363633

[23] Jeon BG Kim HJ Jung KH Kim SW Park JS Kim KH Prolonged operative time in laparoscopic appendectomy: predictive factors and outcomes. Int J Surg. (2016) 36(Pt A):225–32. 10.1016/j.ijsu.2016.10.035 27794471

[24] Fields AC Lu P Palenzuela DL Bleday R Goldberg JE Irani J Does retrieval bag use during laparoscopic appendectomy reduce postoperative infection? Surgery. (2019) 165(5):953–7. 10.1016/j.surg.2018.11.012 30591378

[25] Kossenas K Kouzeiha R Moutzouri O Georgopoulos F. Single-incision versus conventional laparoscopic appendectomy in adults: a systematic review and meta-analysis of randomized controlled trials. Updates Surg. (2025) 77(2):287–96. 10.1007/s13304-025-02112-5 39904954 PMC11961530

[26] Wu S Shen Y Wang J Wei J Chen X. Conventional three-port laparoscopic appendectomy versus transumbilical and suprapubic single-incision laparoscopic appendectomy using only conventional laparoscopic instruments. Langenbecks Arch Surg. (2022) 407(8):3623–9. 10.1007/s00423-022-02683-6 36125515 PMC9722820

[27] Karakus OZ Ulusoy O Ates O Hakguder G Olguner M Akgur FM. Conventional single-port laparoscopic appendectomy for complicated appendicitis in children: efficient and cost-effective. J Minim Access Surg. (2016) 12(1):16–21. 10.4103/0972-9941.171958 26917914 PMC4746969

[28] Lee YS Kim JH Moon EJ Kim JJ Lee KH Oh SJ Comparative study on surgical outcomes and operative costs of transumbilical single-port laparoscopic appendectomy versus conventional laparoscopic appendectomy in adult patients. Surg Laparosc Endosc Percutan Tech. (2009) 19(6):493–6. 10.1097/SLE.0b013e3181c15493 20027094

[29] Agaba EA Rainville H Ikedilo O Vemulapali P. Incidence of port-site incisional hernia after single-incision laparoscopic surgery. JSLS. (2014) 18(2):204–10. 10.4293/108680813X13693422518317 24960483 PMC4035630

